# Publisher Correction: miRNA-mediated gene silencing in *Drosophila* larval development involves GW182-dependent and independent mechanisms

**DOI:** 10.1038/s44318-024-00287-y

**Published:** 2024-10-31

**Authors:** Eriko Matsuura-Suzuki, Kaori Kiyokawa, Shintaro Iwasaki, Yukihide Tomari

**Affiliations:** 1 Institute for Quantitative Biosciences, The University of Tokyo, Bunkyo-ku, Tokyo, 113-0032 Japan; 2 RNA Systems Biochemistry Laboratory, RIKEN Cluster for Pioneering Research, Wako, Saitama, 351-0198 Japan; 3 Institute of Industrial Science, The University of Tokyo, Meguro-ku, Tokyo, 153-8505 Japan; 4 Department of Computational Biology and Medical Sciences, Graduate School of Frontier Sciences, The University of Tokyo, Bunkyo-ku, Tokyo, 113-0032 Japan

## Abstract

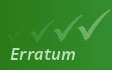

C**orrection to:** *The EMBO Journal* (2024). 10.1038/s44318-024-00249-4 | Published online 25 September 2024

The authors notified the journal of a typographical error in the synopsis text, which occurred prior to the export of the final publication files to the publisher.

**The synopsis text is corrected**.

The synopsis text is corrected from:

GW128 is believed to be essential for miRNA-dependent gene silencing by recruiting the CCR4-NOT deadenylase complex to the RISC-bound mRNA target. This study of *gw182*-deficient flies shows that GW128 is dispensable for miRNA-mediated gene repression during early larval stages, but required for successful completion of larval development.

To: (Changes in bold)

**GW182** is believed to be essential for miRNA-dependent gene silencing by recruiting the CCR4-NOT deadenylase complex to the RISC-bound mRNA target. This study of *gw182*-deficient flies shows that **GW182** is dispensable for miRNA-mediated gene repression during early larval stages, but required for successful completion of larval development.

The original article has been corrected.

