# Supplementary figures and images for: miRNA-mediated gene silencing in Drosophila larval development involves GW182-dependent and independent mechanisms
Source: EMBO J. 2024 Sep 25;43(23):19. doi: 10.1038/s44318-024-00249-4 (PMC11612316; doi:10.1038/s44318-024-00249-4)

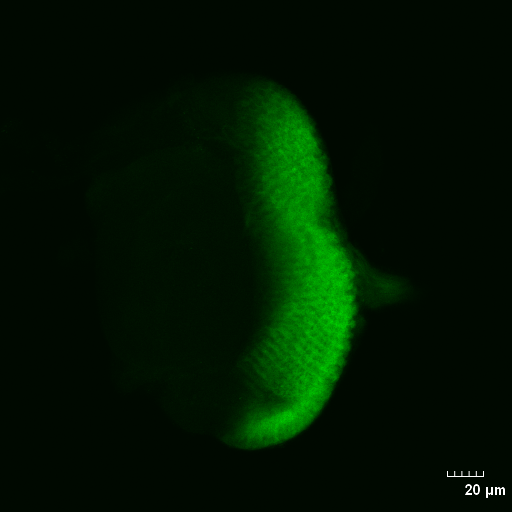

Supplement: Supplementary file 2 — Source data Fig. 1 [file 44318_2024_249_MOESM2_ESM.zip › Figure 1/1C/Mutated_gmrG4/Mutated_gmrG4_GFP.tif]

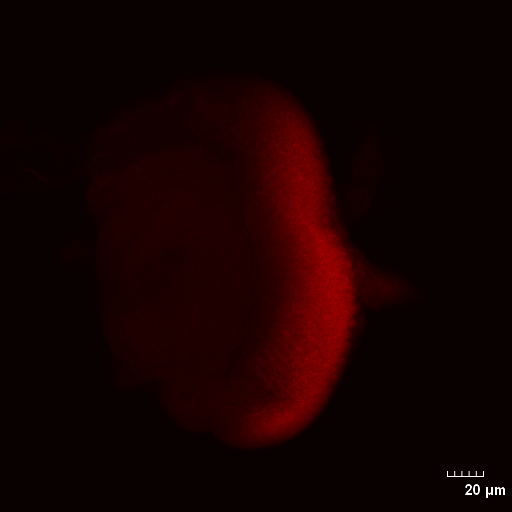

Supplement: Supplementary file 2 — Source data Fig. 1 [file 44318_2024_249_MOESM2_ESM.zip › Figure 1/1C/Mutated_gmrG4/Mutated_gmrG4_mCherry.tif]

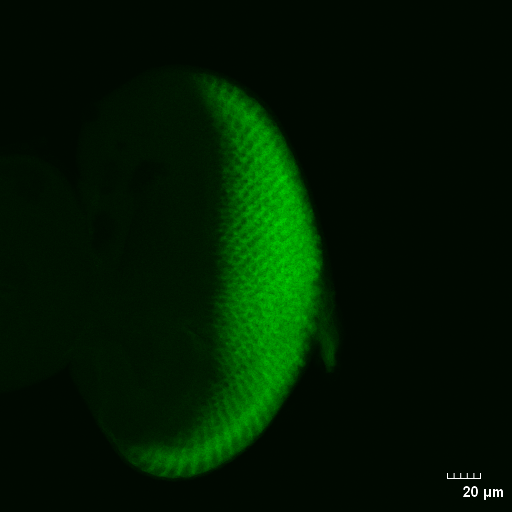

Supplement: Supplementary file 2 — Source data Fig. 1 [file 44318_2024_249_MOESM2_ESM.zip › Figure 1/1C/Mutated_gmrG4_miR-8/Mutated_gmrG4_miR-8_GFP.tif]

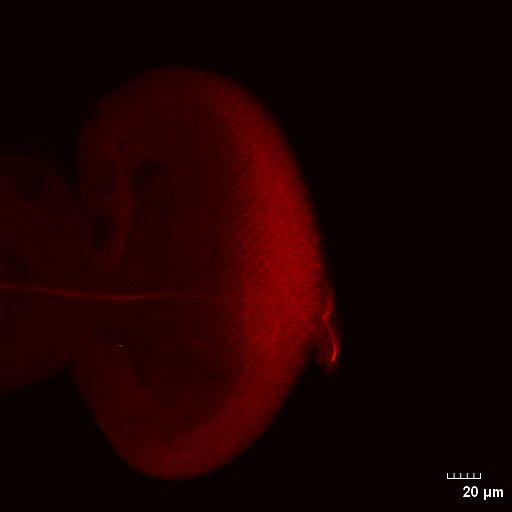

Supplement: Supplementary file 2 — Source data Fig. 1 [file 44318_2024_249_MOESM2_ESM.zip › Figure 1/1C/Mutated_gmrG4_miR-8/Mutated_gmrG4_miR-8_mCherry.tif]

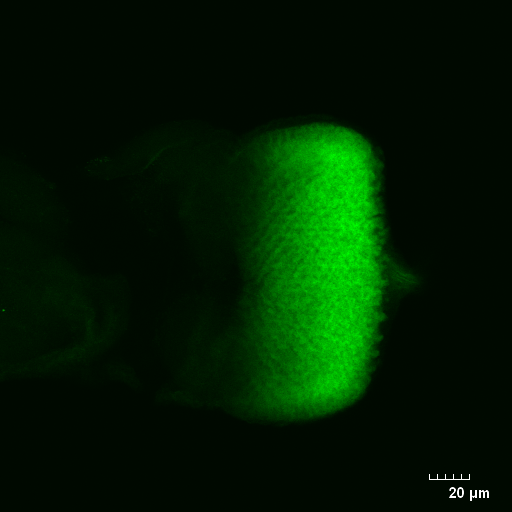

Supplement: Supplementary file 2 — Source data Fig. 1 [file 44318_2024_249_MOESM2_ESM.zip › Figure 1/1C/Sensor_gmr-G4/Sensor_gmr-G4_GFP.tif]

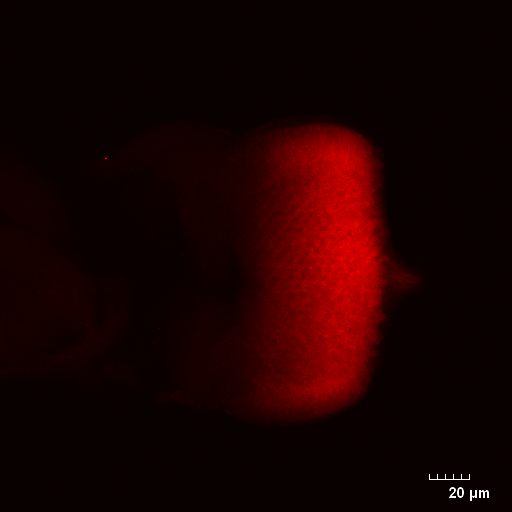

Supplement: Supplementary file 2 — Source data Fig. 1 [file 44318_2024_249_MOESM2_ESM.zip › Figure 1/1C/Sensor_gmr-G4/Sensor_gmr-G4_mCherry.tif]

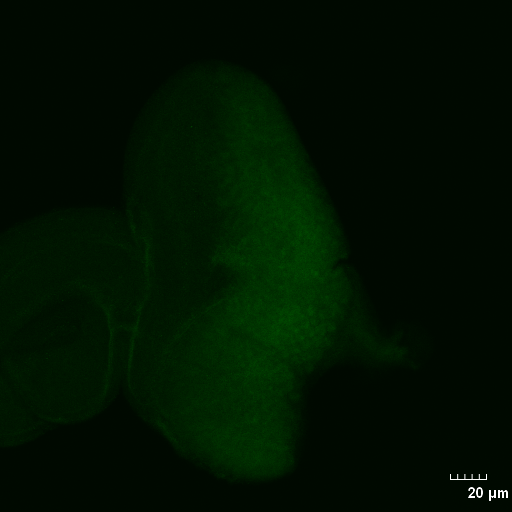

Supplement: Supplementary file 2 — Source data Fig. 1 [file 44318_2024_249_MOESM2_ESM.zip › Figure 1/1C/Sensor_gmrG4_miR-8/Sensor_gmrG4_miR-8_GFP.tif]

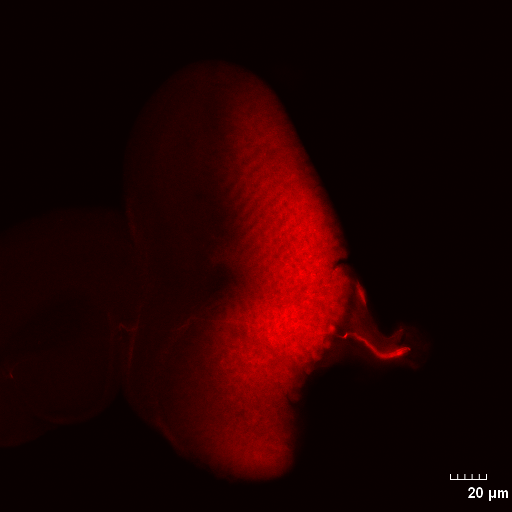

Supplement: Supplementary file 2 — Source data Fig. 1 [file 44318_2024_249_MOESM2_ESM.zip › Figure 1/1C/Sensor_gmrG4_miR-8/Sensor_gmrG4_miR-8_mCherry.tif]

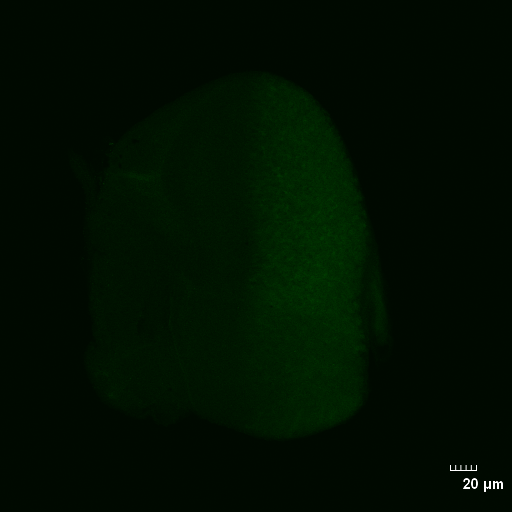

Supplement: Supplementary file 3 — Source data Fig. 2 [file 44318_2024_249_MOESM3_ESM.zip › Figure 2/2A/Sensor_miR-8/8A_miR-8_GFP.tif]

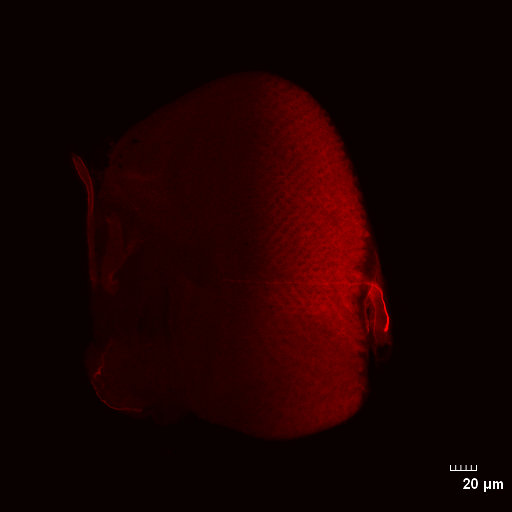

Supplement: Supplementary file 3 — Source data Fig. 2 [file 44318_2024_249_MOESM3_ESM.zip › Figure 2/2A/Sensor_miR-8/8A_miR-8_mCherry.tif]

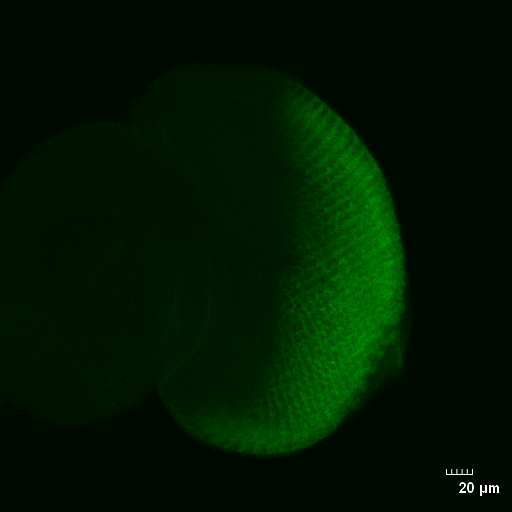

Supplement: Supplementary file 3 — Source data Fig. 2 [file 44318_2024_249_MOESM3_ESM.zip › Figure 2/2A/Sensor_miR-8_ago-IR/Sensor_miR-8_ago1-IR_GFP.tif]

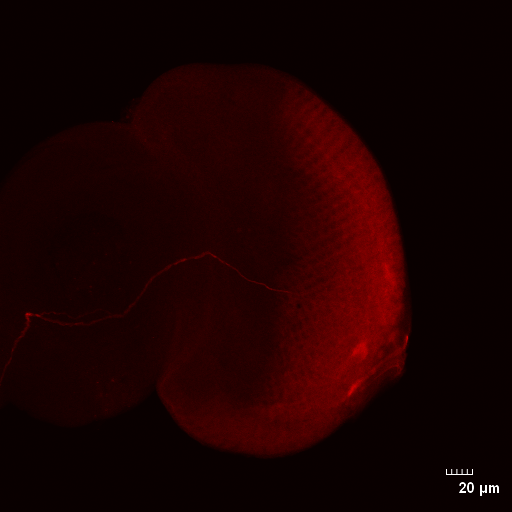

Supplement: Supplementary file 3 — Source data Fig. 2 [file 44318_2024_249_MOESM3_ESM.zip › Figure 2/2A/Sensor_miR-8_ago-IR/Sensor_miR-8_ago1-IR_mCherry.tif]

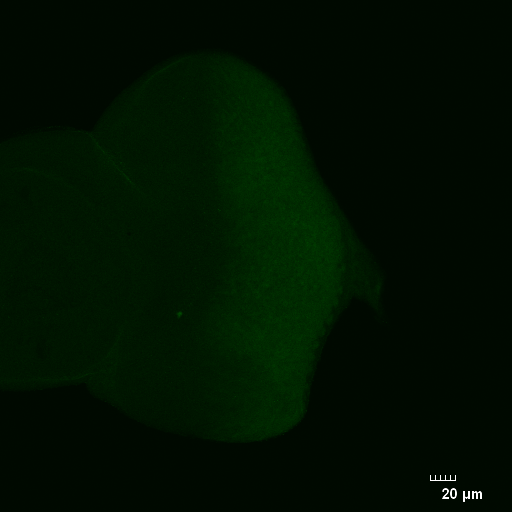

Supplement: Supplementary file 3 — Source data Fig. 2 [file 44318_2024_249_MOESM3_ESM.zip › Figure 2/2A/Sensor_miR-8_gw-IR(1)/Sensor_miR-8_gw-IR(1)_GFP.tif]

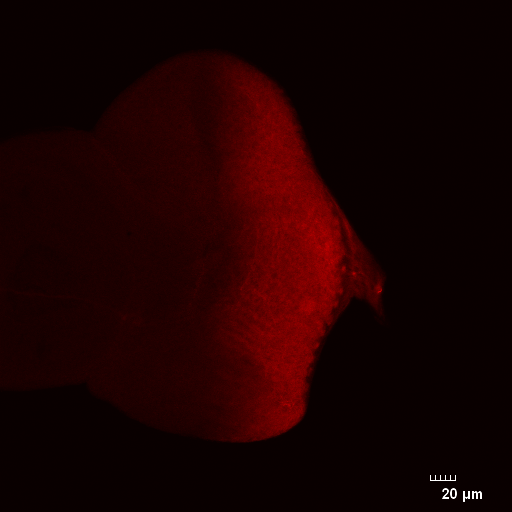

Supplement: Supplementary file 3 — Source data Fig. 2 [file 44318_2024_249_MOESM3_ESM.zip › Figure 2/2A/Sensor_miR-8_gw-IR(1)/Sensor_miR-8_gw-IR(1)_mCherry.tif]

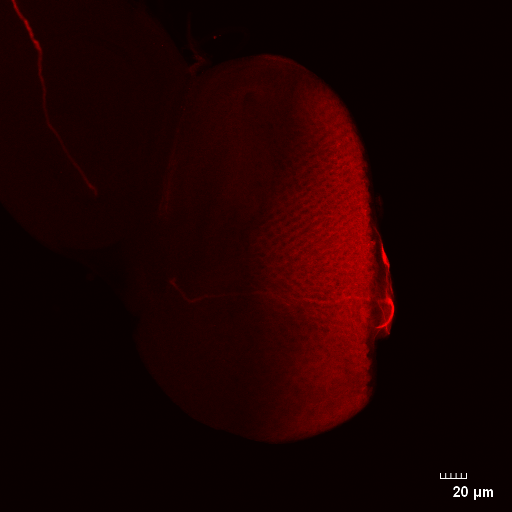

Supplement: Supplementary file 3 — Source data Fig. 2 [file 44318_2024_249_MOESM3_ESM.zip › Figure 2/2A/Sensor_miR-8_gw-IR(2)/Senosor_miR-8_gw-IR(2)_mCherry.tif]

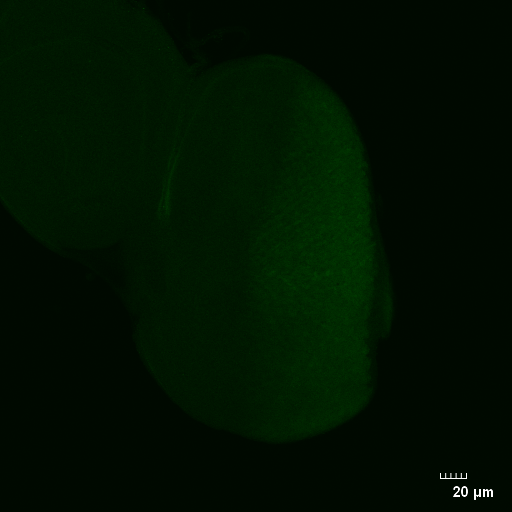

Supplement: Supplementary file 3 — Source data Fig. 2 [file 44318_2024_249_MOESM3_ESM.zip › Figure 2/2A/Sensor_miR-8_gw-IR(2)/Sensor_miR-8_gw-IR(2)_GFP.tif]

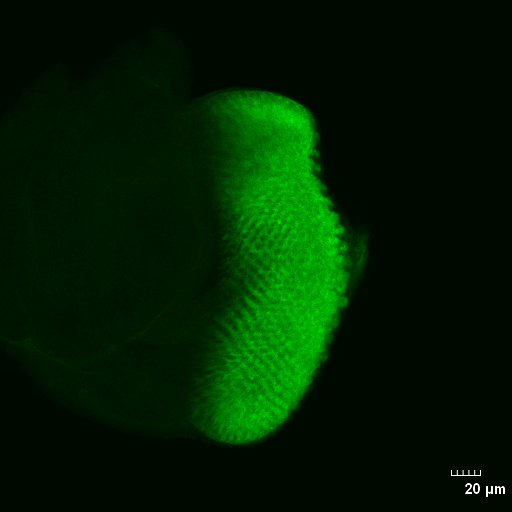

Supplement: Supplementary file 3 — Source data Fig. 2 [file 44318_2024_249_MOESM3_ESM.zip › Figure 2/2B/Mutated_miR-8/Mutated_miR-8_GFP.tif]

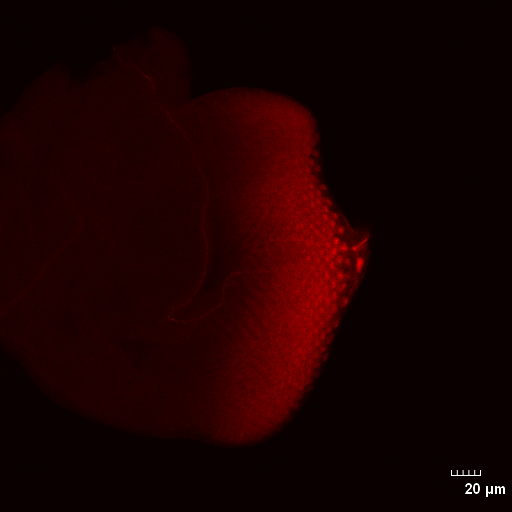

Supplement: Supplementary file 3 — Source data Fig. 2 [file 44318_2024_249_MOESM3_ESM.zip › Figure 2/2B/Mutated_miR-8/Mutated_miR-8_mCherry.tif]

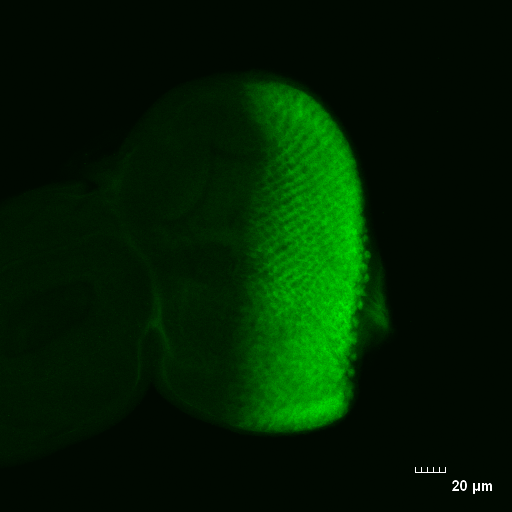

Supplement: Supplementary file 3 — Source data Fig. 2 [file 44318_2024_249_MOESM3_ESM.zip › Figure 2/2B/Mutated_miR-8_ago1-IR/Mutated_miR-8_ago1-IR_GFP.tif]

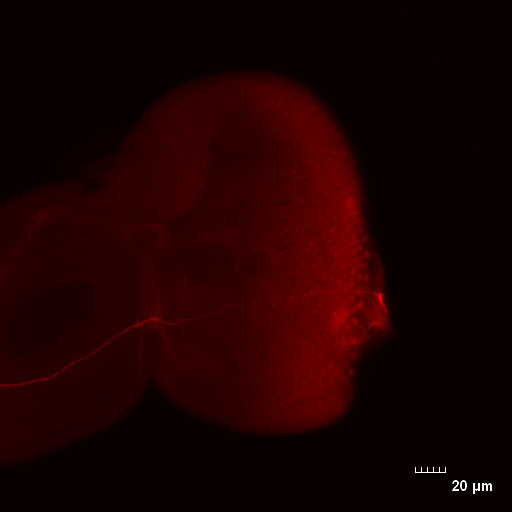

Supplement: Supplementary file 3 — Source data Fig. 2 [file 44318_2024_249_MOESM3_ESM.zip › Figure 2/2B/Mutated_miR-8_ago1-IR/Mutated_miR-8_ago1-IR_mCherry.tif]

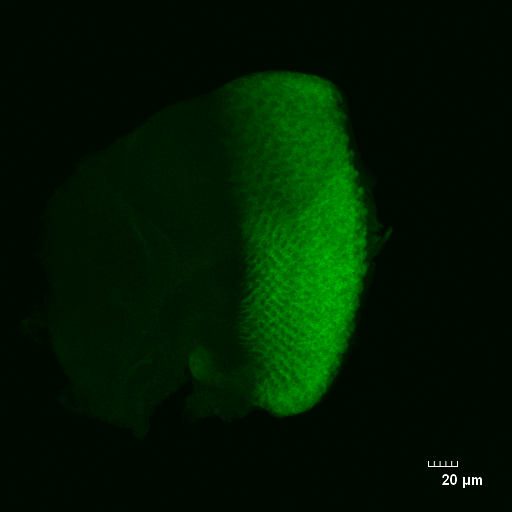

Supplement: Supplementary file 3 — Source data Fig. 2 [file 44318_2024_249_MOESM3_ESM.zip › Figure 2/2B/Mutated_miR-8_gw-IR(1)/Mutated_miR-8_gw-IR(1)_GFP.tif]

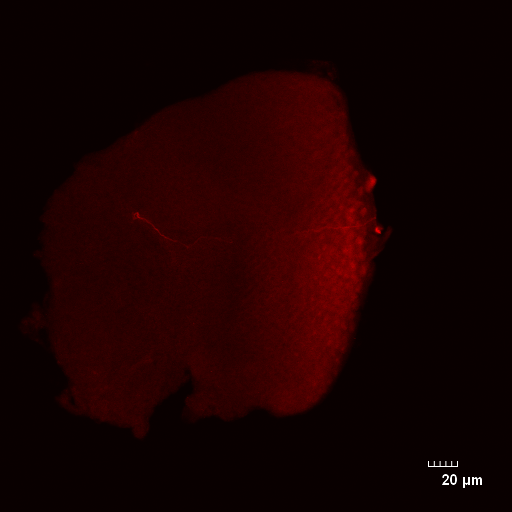

Supplement: Supplementary file 3 — Source data Fig. 2 [file 44318_2024_249_MOESM3_ESM.zip › Figure 2/2B/Mutated_miR-8_gw-IR(1)/Mutated_miR-8_gw-IR(1)_mCherry.tif]

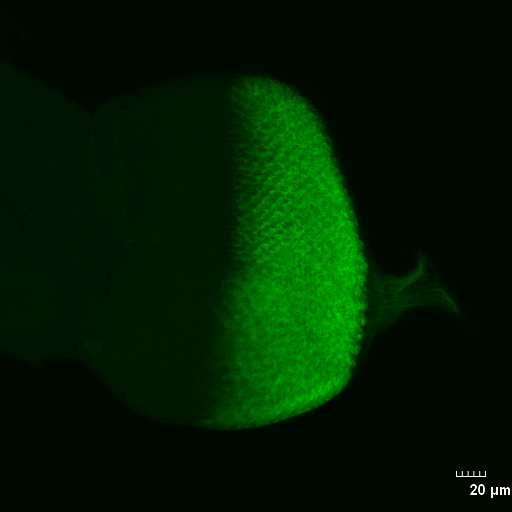

Supplement: Supplementary file 3 — Source data Fig. 2 [file 44318_2024_249_MOESM3_ESM.zip › Figure 2/2B/Mutated_miR-8_gw-IR(2)/Mutated_miR-8_gw-IR(2)_GFP.tif]

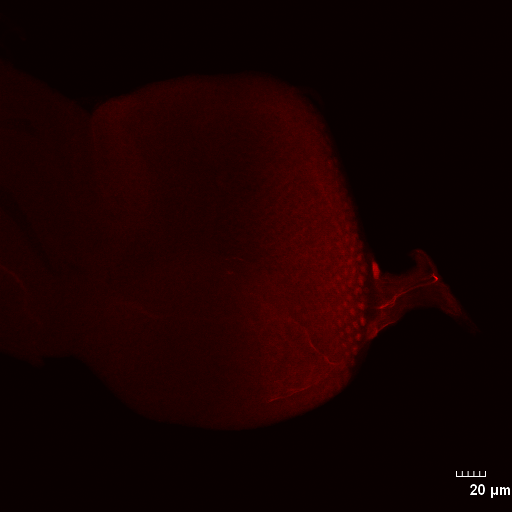

Supplement: Supplementary file 3 — Source data Fig. 2 [file 44318_2024_249_MOESM3_ESM.zip › Figure 2/2B/Mutated_miR-8_gw-IR(2)/Mutated_miR-8_gw-IR(2)_mCherry.tif]

Figure 3E

Description of original data

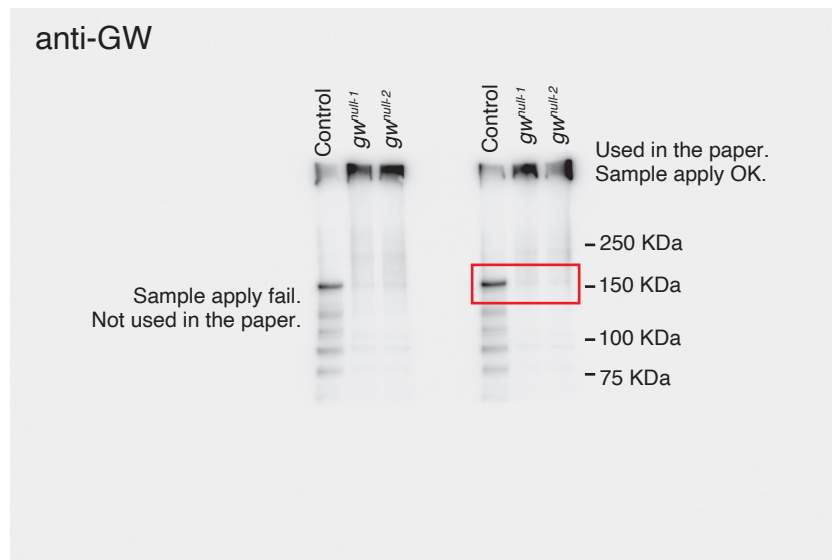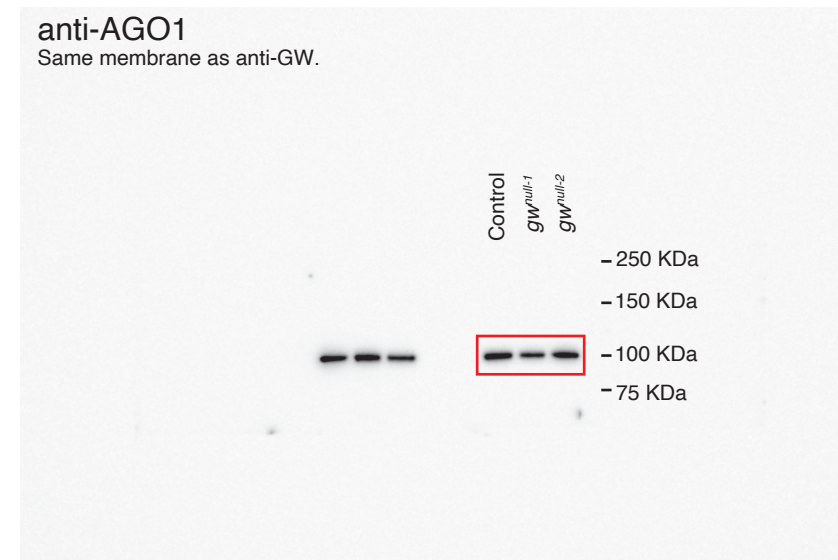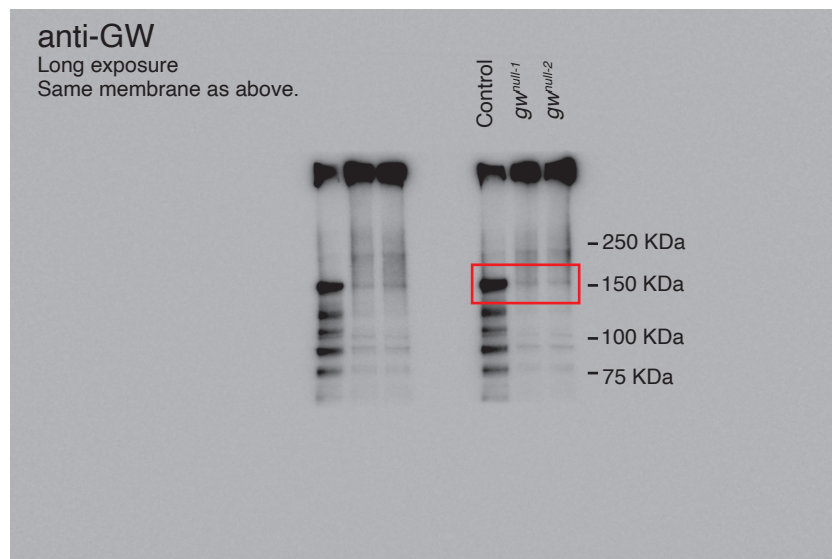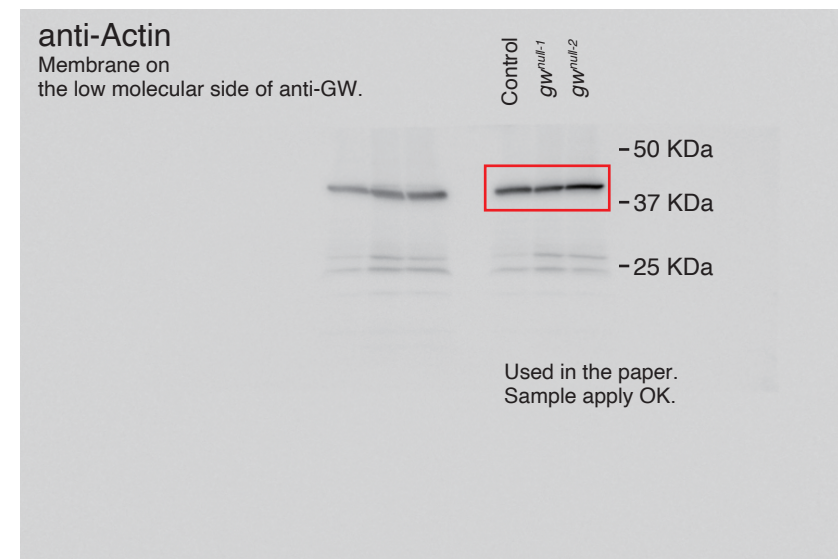

Supplement: Supplementary file 4 — Source data Fig. 3 [file 44318_2024_249_MOESM4_ESM.zip › Figure 3/3E/Description of Figure 3E.pdf]

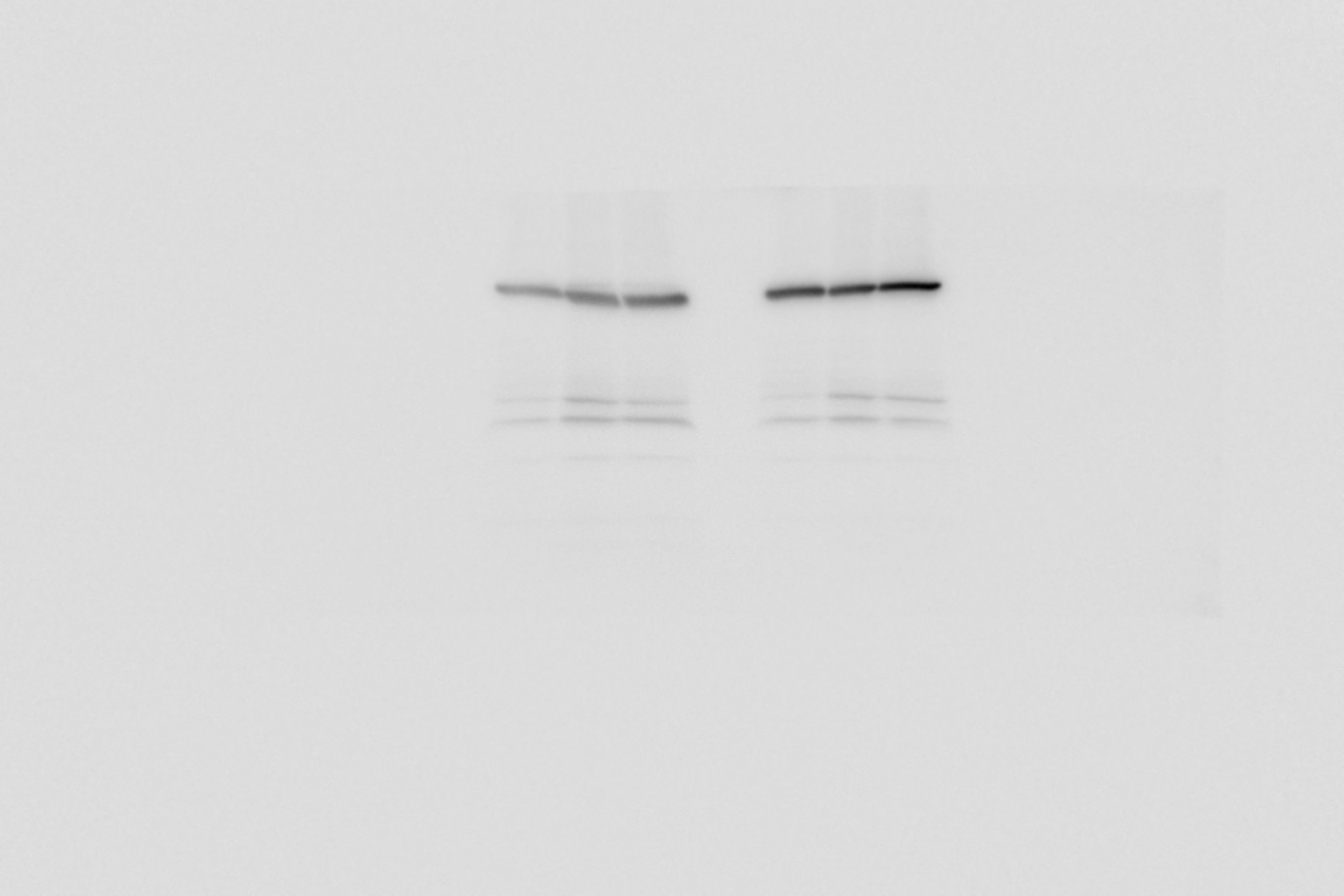

Supplement: Supplementary file 4 — Source data Fig. 3 [file 44318_2024_249_MOESM4_ESM.zip › Figure 3/3E/SourceData_Figure 3E_anti-actin.tif]

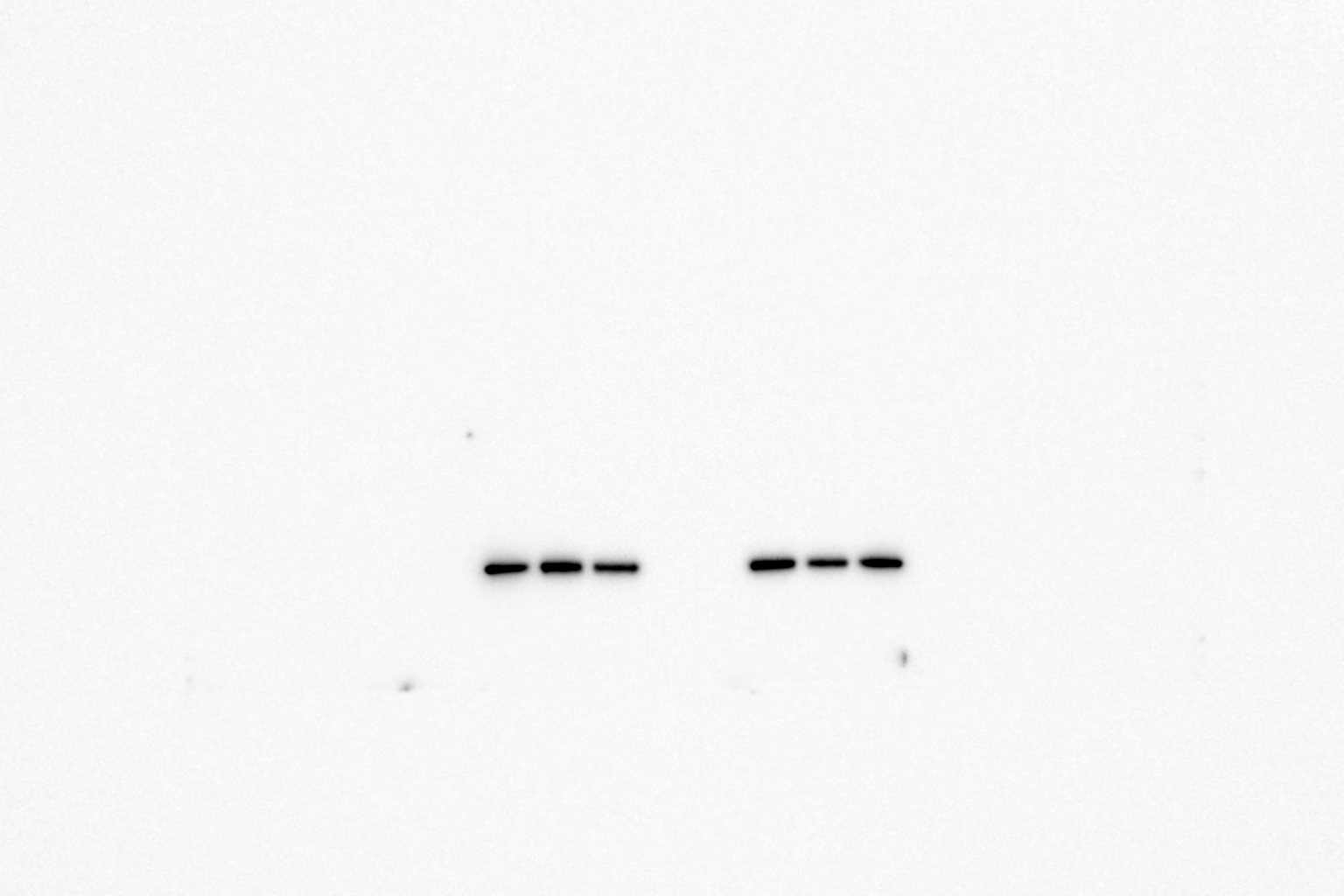

Supplement: Supplementary file 4 — Source data Fig. 3 [file 44318_2024_249_MOESM4_ESM.zip › Figure 3/3E/SourceData_Figure 3E_anti-AGO1.tif]

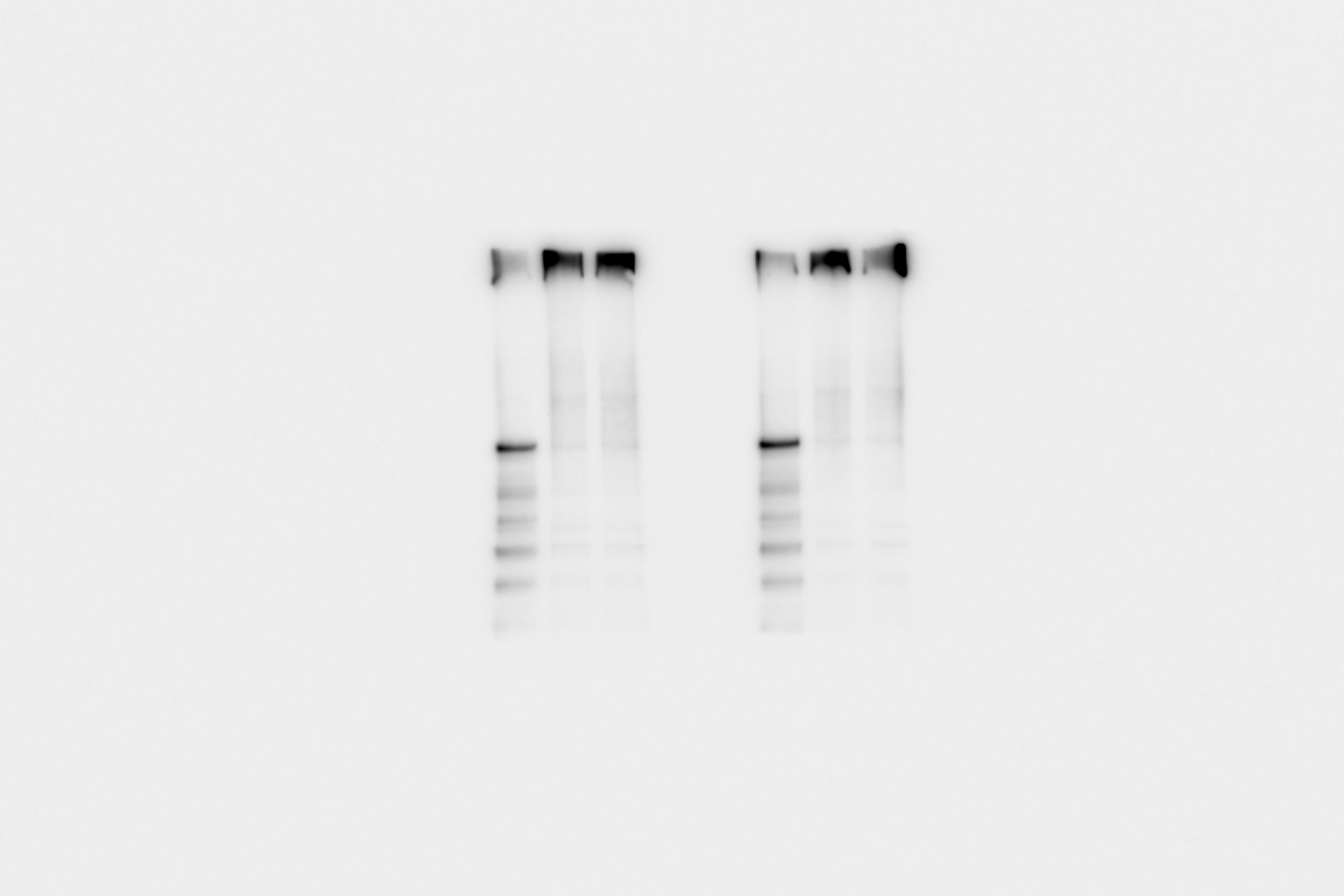

Supplement: Supplementary file 4 — Source data Fig. 3 [file 44318_2024_249_MOESM4_ESM.zip › Figure 3/3E/SourceData_Figure 3E_anti-GW.tif]

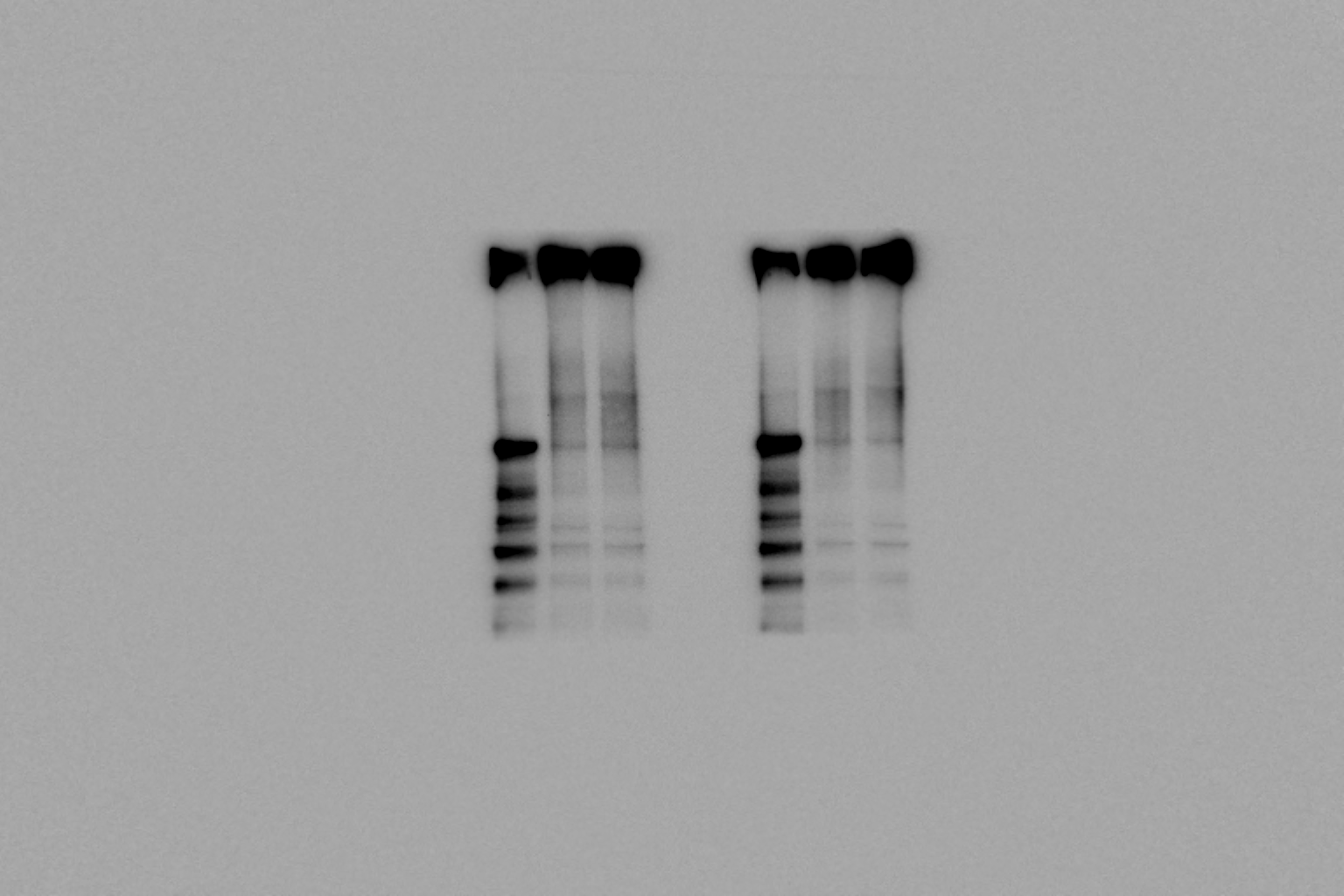

Supplement: Supplementary file 4 — Source data Fig. 3 [file 44318_2024_249_MOESM4_ESM.zip › Figure 3/3E/SourceData_Figure 3E_anti-GW_longExposure.tif]

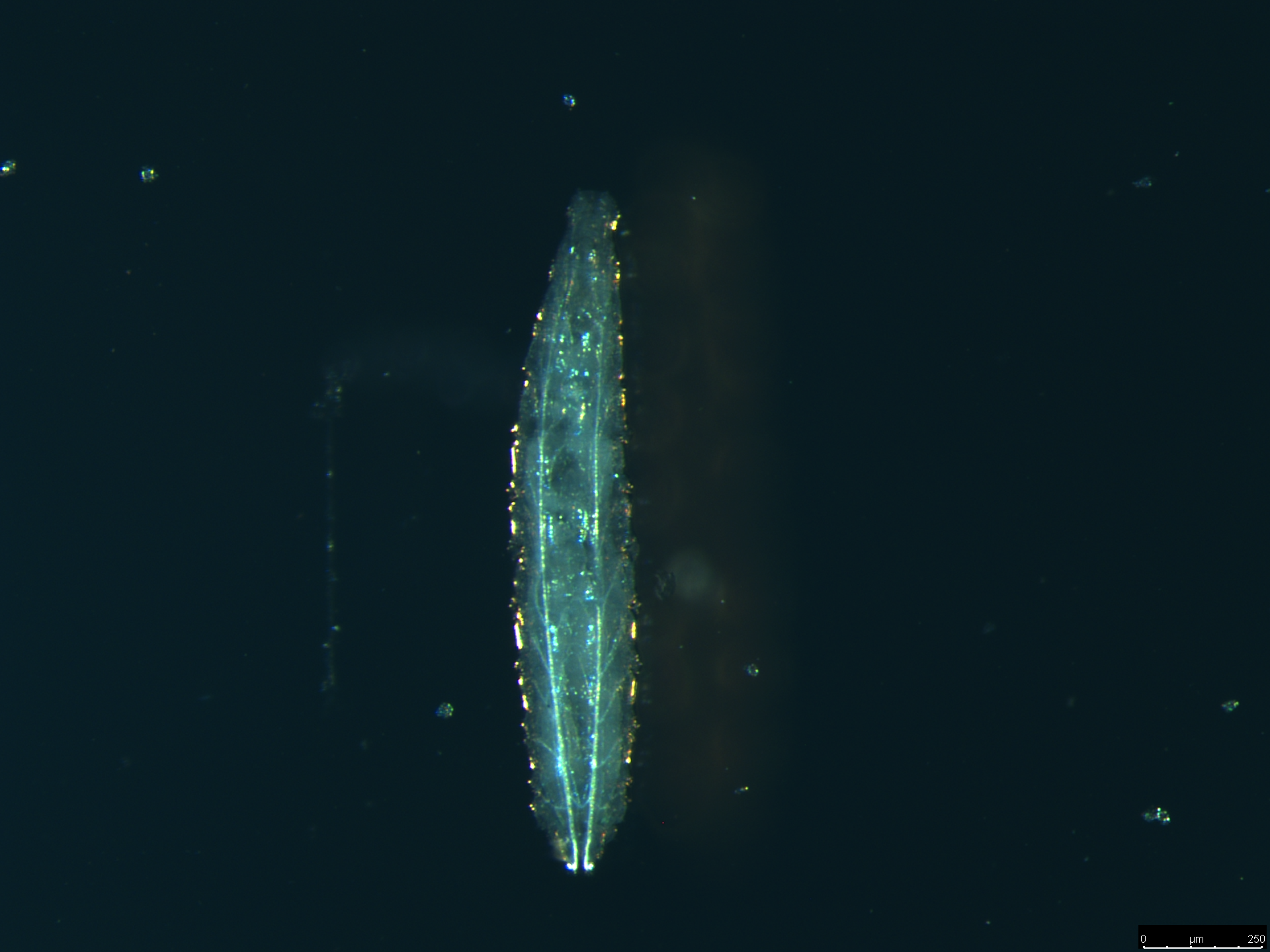

Supplement: Supplementary file 7 — Source data Fig. 7 [file 44318_2024_249_MOESM7_ESM.zip › Figure 7/7A/gw null-2.tif]

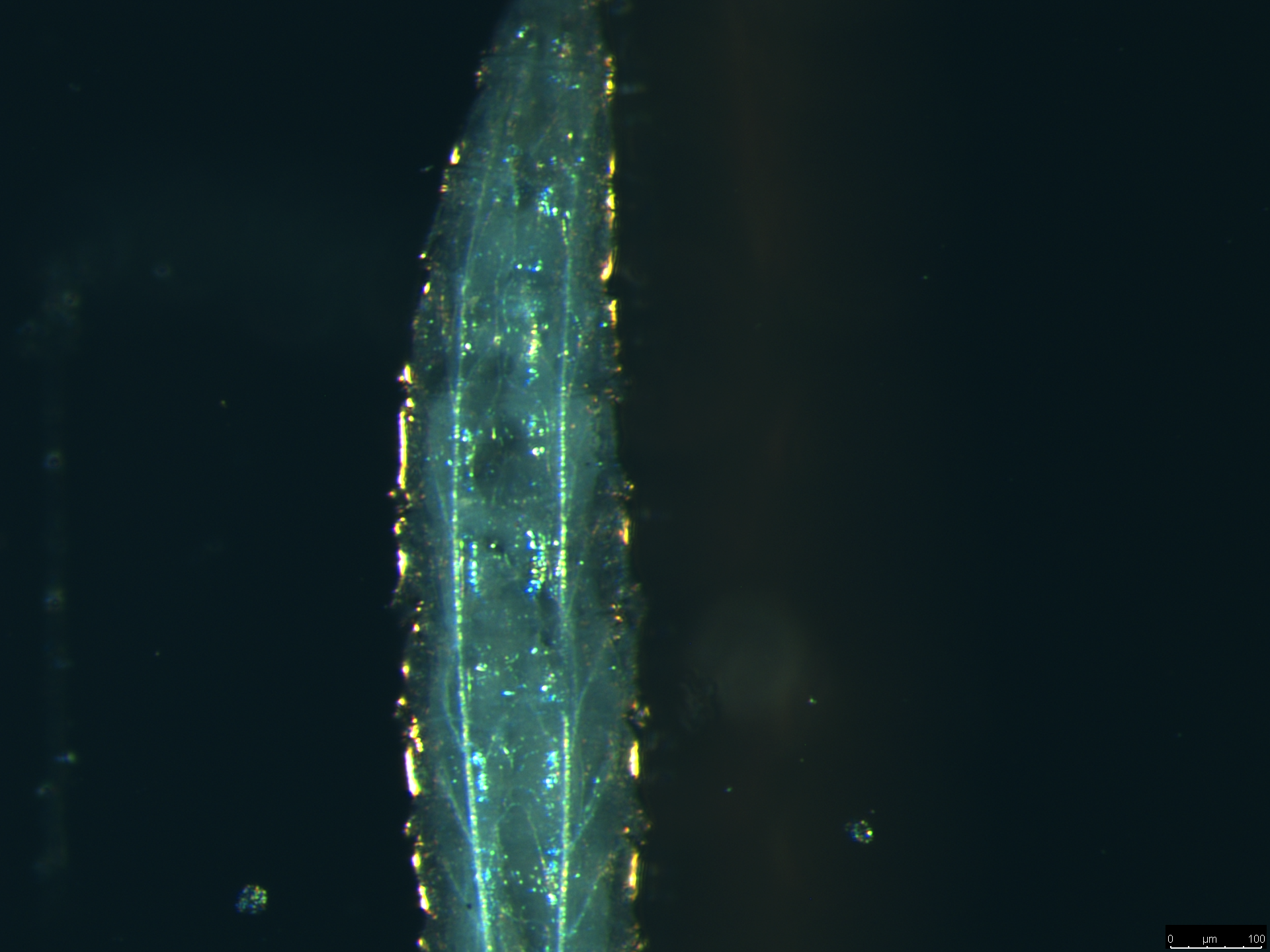

Supplement: Supplementary file 7 — Source data Fig. 7 [file 44318_2024_249_MOESM7_ESM.zip › Figure 7/7A/gw null-2_enlarged.tif]

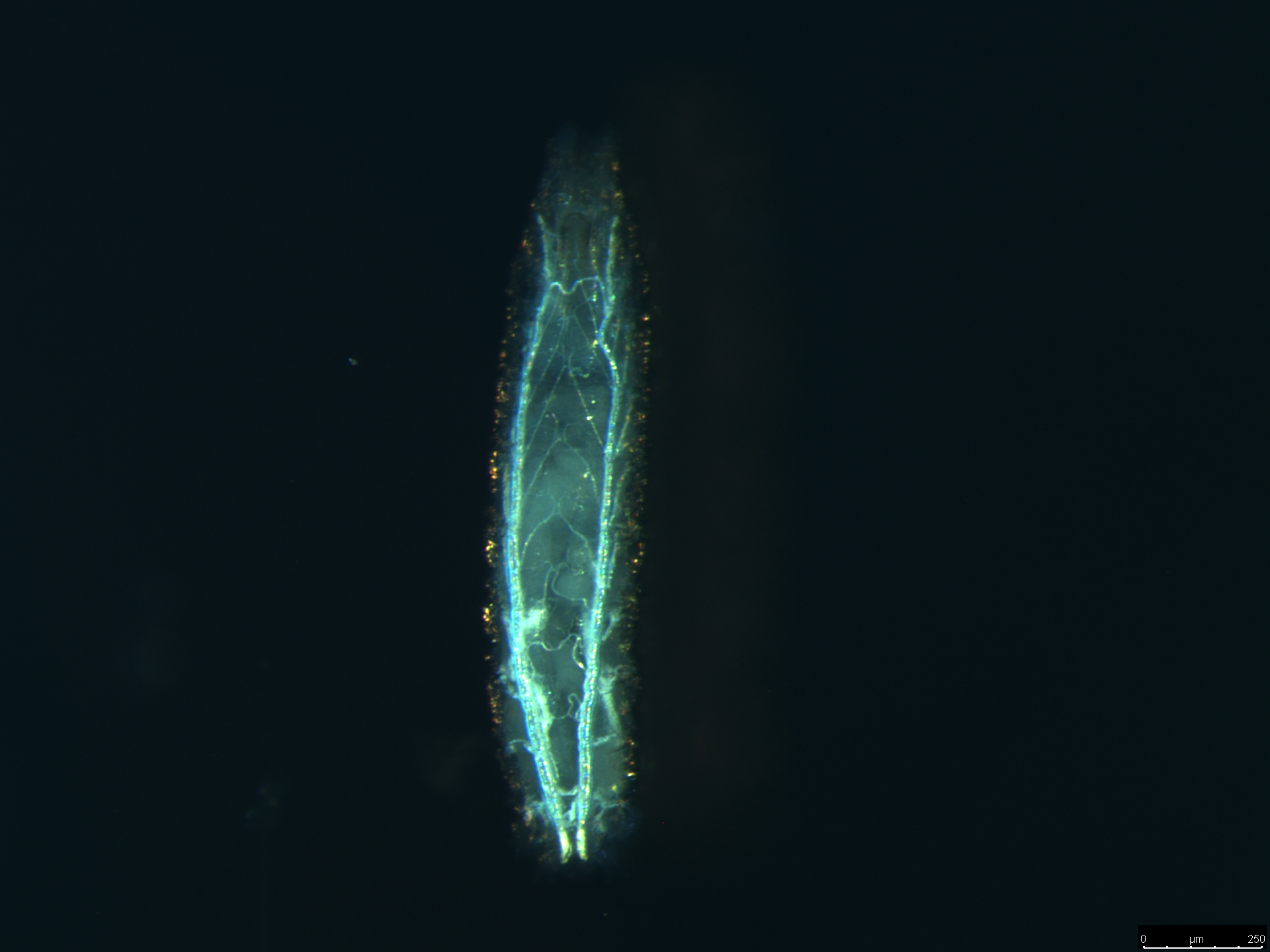

Supplement: Supplementary file 7 — Source data Fig. 7 [file 44318_2024_249_MOESM7_ESM.zip › Figure 7/7A/yw.tif]
