# Supplementary material for: miRNA-mediated gene silencing in Drosophila larval development involves GW182-dependent and independent mechanisms
Source: EMBO J. 2024 Sep 25;43(23):19. doi: 10.1038/s44318-024-00249-4 (PMC11612316; doi:10.1038/s44318-024-00249-4)
Supplement: Supplementary file 8 — Expanded View Figures [file 44318_2024_249_MOESM8_ESM.pdf]

Expanded View Figures

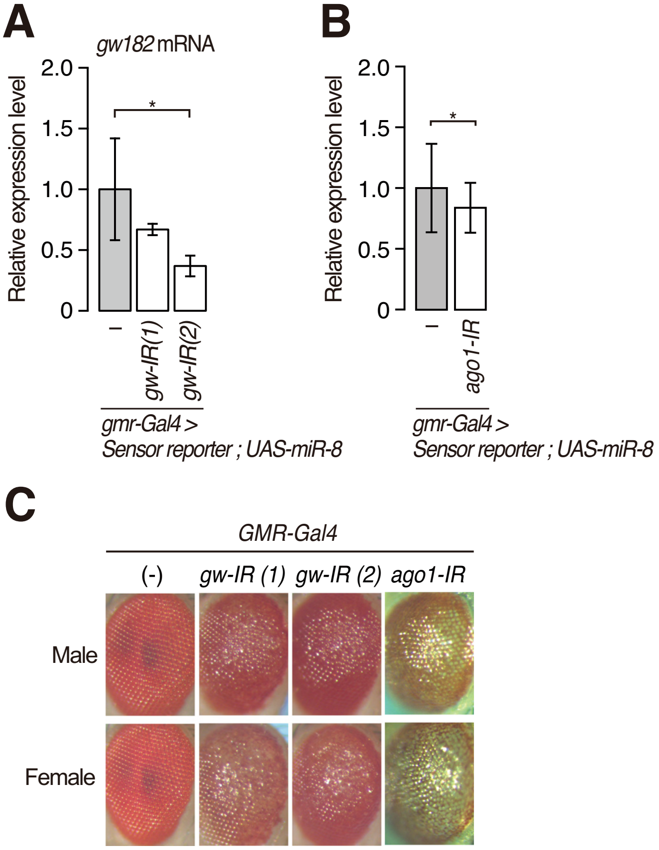

Figure EV1. Quantification of *gw182* mRNA expression level in *gw182* knockdown flies.

(**A**) Quantification of *gw182* mRNA expression. The *gw182* mRNA levels of two independent *gw182*-IR-expressing strains, *gw-IR(1)* and *gw-IR(2)*, at the third instar larval stage were measured by qRT-PCR. *rp49* was used as a reference. The relative expression levels against the control are shown as mean ± SD. Error bars represent standard derivation from three independent experiments. *P* values were calculated by one-way ANOVA with the post hoc Tukey HSD test. *$P = 0.047$. (**B**) Quantification of *ago1* mRNA expression. The *ago1* mRNA levels of *ago1-IR*-expressing strains at the third instar larval stage were measured by qRT-PCR. rp49 was used as a reference. The relative expression levels against the control are shown as mean ± SD. Error bars represent standard derivation from three independent experiments. *P* values were calculated by Student's *t* test (unpaired, two-sided). *$P = 0.538$. (**C**) Rough eye phenotype by *gw182 or ago1* knockdown. The eye phenotypes of two independent *gw182*-IR-expressing strains, *gw-IR(1)* and *gw-IR(2)*, and *ago1-IR*-expressing strain at the adult stage were observed. *gmr-G4* strain was used as a control. Genotypes: *GMR-GAL4/+*, *GMR-GAL4,GW182-IR(1)/+*, *GMR-GAL4,GW182-IR(2)/+*, *GMR-GAL4,ago1-IR/+*.

© The Author(s)

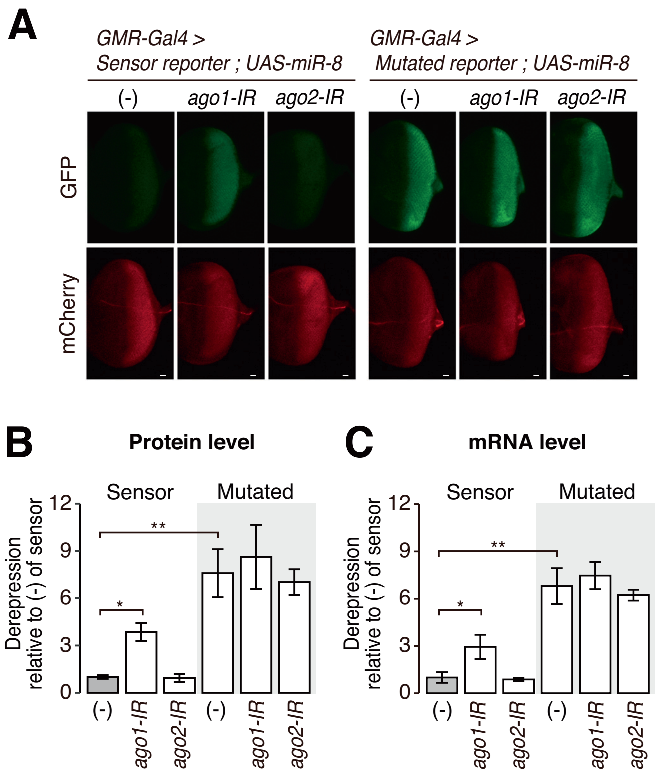

Figure EV2. Ago2 is not essential for miR-8-mediated silencing of the reporter in eye discs.

(**A**) Fluorescence microscopy images for the Sensor reporter (**A**) and the Mutated reporter (**B**) upon miR-8 overexpression. *ago1* or *ago2* was knocked down by corresponding IR in the third instar larval eye disc. Scale bar: 20 µm. (**B**) Quantified derepression at the protein level in Fig. EV1A. The GFP fluorescence level was normalized to the mCherry fluorescence level, and the derepression levels are shown relative to the negative control without IR expression (-) of the Sensor reporter or the Mutated reporter as mean ± SD. Error bars represent standard derivation from 10 independent eye discs for each sample. *P* values were calculated by one-way ANOVA with the post hoc Tukey HSD test. $^*P = 0.78e\text{-}5$; $^{**}P < 1.00e\text{-}7$. (**C**) Quantified derepression at the mRNA level in the Sensor and Mutated reporters. GFP mRNA level was normalized to mCherry mRNA level, and the derepression levels are relative to the negative control (-) of the Sensor reporter, shown as mean ± SD. Error bars represent standard derivation from 3 independent experiments. *P* values were calculated by one-way ANOVA with the post hoc Tukey HSD test. $^*P = 4.31e\text{-}2$; $^{**}P = 3.20e\text{-}6$. Genotypes: *GMR-GAL4/UAS-Sensor reporter; UAS-miR-8/+* ($n = 10$), *GMR-GAL4,ago1-IR/UAS-Sensor reporter; UAS-miR-8/+* ($n = 10$), *GMR-GAL4,ago1-IR/UAS-Sensor reporter; UAS-miR-8/ago2-IR* ($n = 10$), *GMR-GAL4/UAS-Mutated reporter; UAS-miR-8/+* ($n = 10$), *GMR-GAL4,ago1-IR/UAS-Mutated reporter; UAS-miR-8/+* ($n = 10$), *GMR-GAL4,ago1-IR/UAS-Mutated reporter; UAS-miR-8/ago2-IR* ($n = 10$). The numbers (n) are for Fig. EV2D.

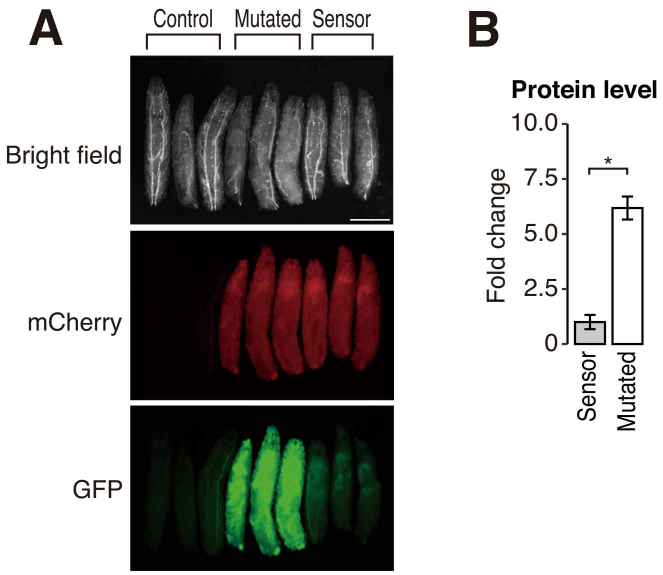

Figure EV3. The Sensor reporter monitors endogenous miR-8 silencing activity at the larval stage.

(**A**) Fluorescence microscopy of the Sensor reporter and the Mutated reporter at first-instar larval stage. Bright field, GFP (green), or mCherry (red) expressions are compared between *yw*, the Sensor reporter strain, or the Mutated reporter strain. *yw* was used as a negative control. Scale bar: 250 µm. (**B**) Quantification of the GFP protein level for Fig. EV3A. The fluorescence GFP levels were normalized to the fluorescence mCherry levels, shown as mean ± SD. Error bars represent standard derivation from three independent larvae for each sample and *P* values were calculated by Student's *t* test (unpaired, two-sided). $^*P < 2.2e\text{-}16$. Genotypes: *yw, miR-8-GAL4/UAS-Sensor reporter* ($n = 42$) and *miR-8-GAL4/UAS-Mutated reporter* ($n = 47$). The numbers (n) are for Fig. EV3B.

 © The Author(s)

A

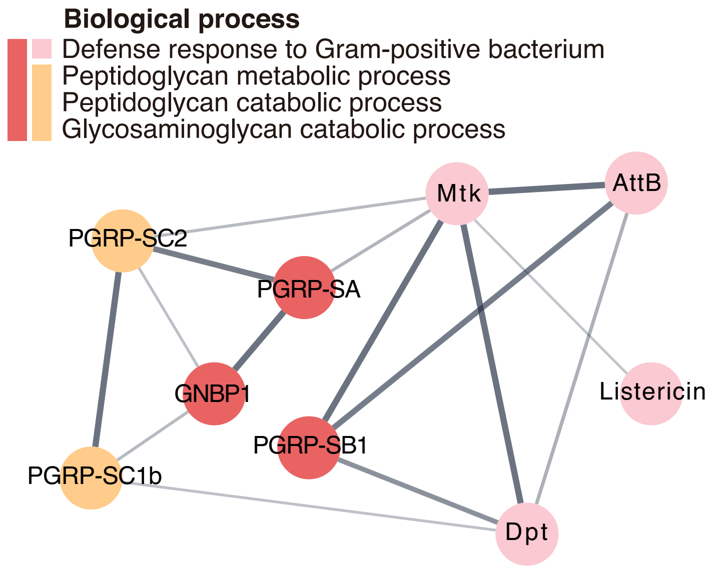
Biological process
Defense response to Gram-positive bacterium
Peptidoglycan metabolic process
Peptidoglycan catabolic process
Glycosaminoglycan catabolic process
Mtk
AttB
PGRP-SC2
PGRP-SA
GNBP1
Listericin
PGRP-SB1
PGRP-SC1b
Dpt

B

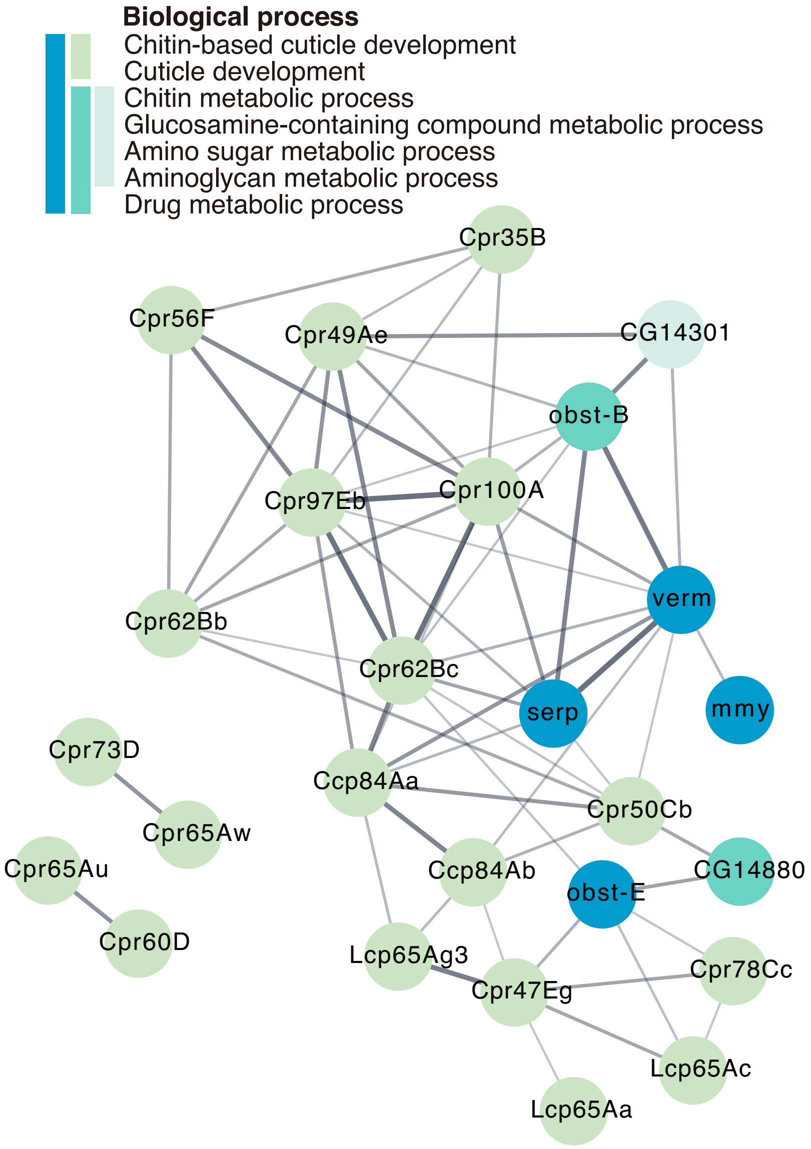
Biological process
Chitin-based cuticle development
Cuticle development
Chitin metabolic process
Glucosamine-containing compound metabolic process
Amino sugar metabolic process
Aminoglycan metabolic process
Drug metabolic process
Cpr35B
Cpr56F
Cpr49Ae
CG14301
obst-B
Cpr97Eb
Cpr100A
verm
Cpr62Bb
Cpr62Bc
serp
mmy
Cpr73D
Cpr65Aw
Ccp84Aa
Cpr50Cb
Cpr65Au
Ccp84Ab
obst-E
CG14880
Cpr60D
Lcp65Ag3
Cpr47Eg
Cpr78Cc
Lcp65Ac
Lcp65Aa

◀ **Figure EV4. Networks of *gw182*-regulated genes and related biological processes.**

(**A**) A protein–protein association network of enriched genes in *gw182*-null mutant and their associated functional classes. Genes classified to defense to gram-positive bacterium, pink; genes classified to peptidoglycan metabolic process, peptidoglycan catabolic process and glycosaminoglycan catabolic process, orange; genes classified to the all above GO terms, red. (**B**) A protein–protein association network of depleted genes in *gw182*-null mutant and their associated functional classes. Genes classified to chitin-based cuticle development and cuticle development, lime green; Genes classified to chitin metabolic process, glucosamine-containing compound metabolic process, amino sugar metabolic process, and aminoglycan metabolic process, light blue; genes classified to GO terms represented by light blue and drug metabolic process, aqua green; genes classified to all above GO terms, aqua blue.

 © The Author(s)

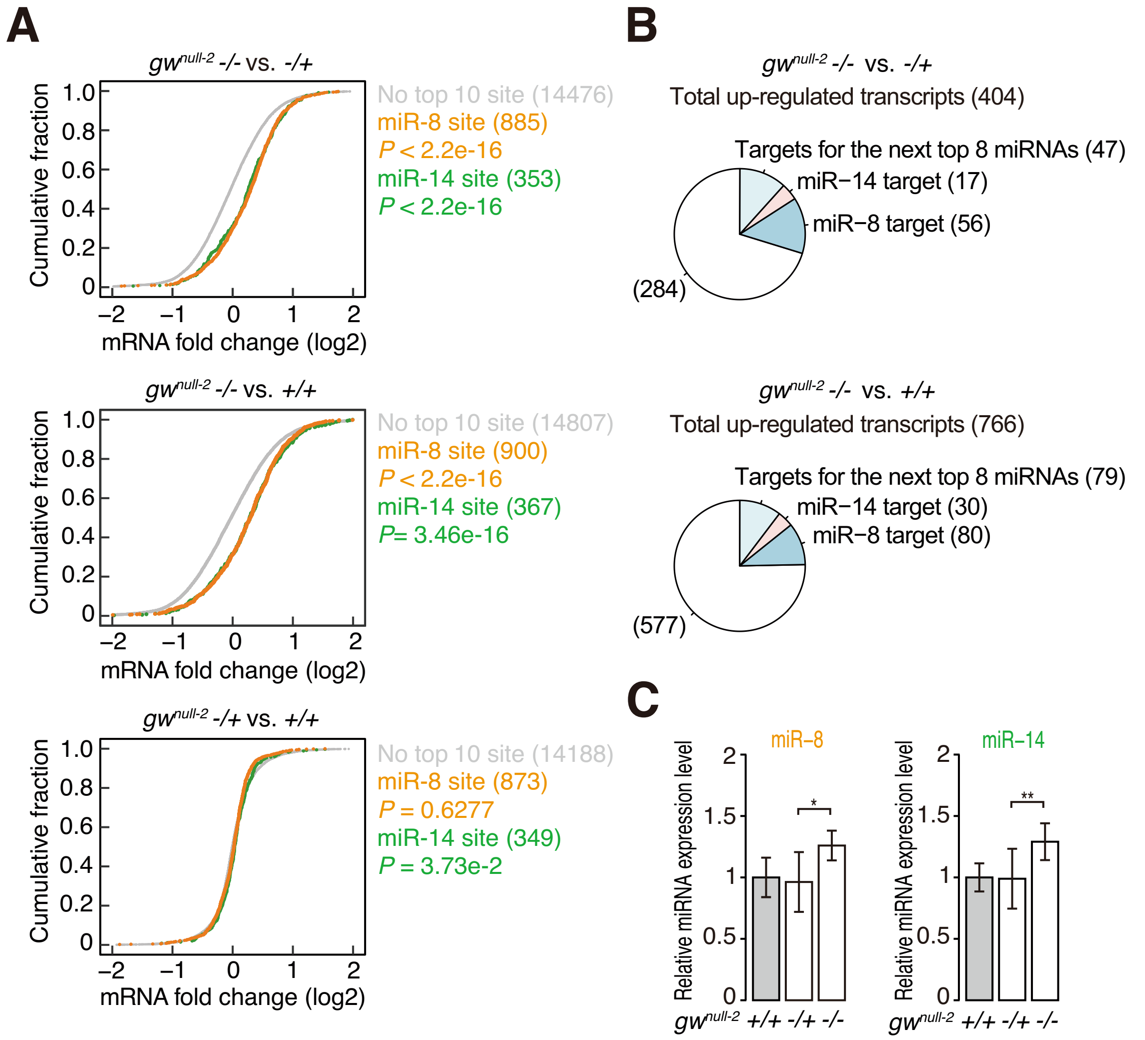

Figure EV5. Depletion of GW182 impaired miRNA-mediated silencing for endogenous miRNA targets at the first-instar larval stage, related to Fig. 5.

(**A**) Upregulation of miRNA targets by knockdown of *gw182*. First-instar larvae of gw^{null-2} homozygote, gw^{null-2} heterozygote or wild type were collected among siblings, and their transcriptome was analyzed by RNA-seq. The fold change values, gw^{null-2} homozygote/gw^{null-2} heterozygote, gw^{null-2} homozygote/wild type, or gw^{null-2} heterozygote/wild type were calculated. The cumulative fractions of the average fold change values of two independent experiments are shown for the following categories: mRNAs without a target site of the top 10 most abundant miRNAs (gray) and mRNAs with predicted target sites for miR-8 (orange) or miR-14 (green), together with their *P* values of the Mann–Whitney *U* test. *P* values are shown in the figure. (**B**) Pie chart showing the percentage of predicted miRNA targets within the transcripts upregulated in gw^{null-2} homozygote compared to gw^{null-2} heterozygote or wild type among siblings. The numbers of transcripts in each category are shown in parentheses. (**C**) Quantification of microRNA expression. The relative expression levels of miR-8 and miR-14 were measured by qRT-PCR from first-instar larvae of gw^{null-2} homozygote, gw^{null-2} heterozygote, or wild type among siblings. *U6 snoRNA* was used as a reference. The data were normalized by the miRNA expression level of wild type, shown as mean ± SD. Error bars represent standard derivation from three independent experiments. *P* values were calculated by Student's *t* test (unpaired, two-sided). **P* = 0.106; ***P* = 0.092.

© The Author(s)
